# Community based intervention to optimize osteoporosis management: randomized controlled trial

**DOI:** 10.1186/1471-2318-10-60

**Published:** 2010-08-27

**Authors:** Patricia M Ciaschini, Sharon E Straus, Lisa R Dolovich, Ron A Goeree, Karen M Leung, Carol R Woods, Greg M Zimmerman, Sumit R Majumdar, Silvana Spadafora, Luke A Fera, Hui N Lee

**Affiliations:** 1 Algoma District Medical Group, Sault Ste. Marie, Canada; 2 Li Ka Shing Knowledge Institute, St. Michael's Hospital, Toronto, Canada; 3 Department of Medicine, University of Toronto, Toronto, Canada; 4 Department of Family Medicine, McMaster University, Hamilton, Canada; 5 Centre for Evaluation of Medicines, St. Joseph's Healthcare, Hamilton, Canada; 6 Program for Assessment of Technology in Health (PATH) Research Institute, St. Joseph's Hospital, Hamilton, Canada; 7 Department of Clinical Epidemiology & Biostatistics, McMaster University, Hamilton, Canada; 8 Department of Physical Therapy, Group Health Centre, Sault Ste. Marie, Canada; 9 Algoma Public Health, Sault Ste. Marie, Canada; 10 Department of Biology, Lake Superior State University, Sault Ste. Marie, USA; 11 Department of Medicine, University of Alberta, Edmonton, Canada; 12 Clinical Research Department, Group Health Centre, Sault Ste. Marie, Canada; 13 Department of Epidemiology & Biostatistics, Schulich School of Medicine and Dentistry, The University of Western Ontario, London, Canada

## Abstract

**Background:**

Osteoporosis-related fractures are a significant public health concern. Interventions that increase detection and treatment of osteoporosis are underutilized. This pragmatic randomised study was done to evaluate the impact of a multifaceted community-based care program aimed at optimizing evidence-based management in patients at risk for osteoporosis and fractures.

**Methods:**

This was a 12-month randomized trial performed in Ontario, Canada. Eligible patients were community-dwelling, aged ≥55 years, and identified to be at risk for osteoporosis-related fractures. Two hundred and one patients were allocated to the intervention group or to usual care. Components of the intervention were directed towards primary care physicians and patients and included facilitated bone mineral density testing, patient education and patient-specific recommendations for osteoporosis treatment. The primary outcome was the implementation of appropriate osteoporosis management.

**Results:**

101 patients were allocated to intervention and 100 to control. Mean age of participants was 71.9 ± 7.2 years and 94% were women. Pharmacological treatment (alendronate, risedronate, or raloxifene) for osteoporosis was increased by 29% compared to usual care (56% [29/52] vs. 27% [16/60]; relative risk [RR] 2.09, 95% confidence interval [CI] 1.29 to 3.40). More individuals in the intervention group were taking calcium (54% [54/101] vs. 20% [20/100]; RR 2.67, 95% CI 1.74 to 4.12) and vitamin D (33% [33/101] vs. 20% [20/100]; RR 1.63, 95% CI 1.01 to 2.65).

**Conclusions:**

A multi-faceted community-based intervention improved management of osteoporosis in high risk patients compared with usual care.

**Trial Registration:**

This trial has been registered with clinicaltrials.gov (ID: NCT00465387)

## Background

Osteoporosis, a chronic condition characterized by low bone mass, microarchitectural deterioration of bone and increased risk of fracture, is a significant public health concern. It affects over 200 million people worldwide [1], an estimated 10 million people in the US [2], 4 million people in the UK [3] and 1.4 million people in Canada [2,4]. Fragility fractures are the clinical consequence of osteoporosis. While vertebral fractures can cause back pain, loss of height and disability [5-7], hip fractures have a more significant impact on quality of life leading to loss of function, and admission to long-term care [8-12]. It is estimated that 1 in 5 people who suffer a hip fracture will die during the first year and less than one third gain their pre-fracture level of function [2]. Moreover, the economic impact of this condition is considerable, with the total acute care cost of osteoporosis estimated to be over $1.3 billion per year in Canada [12], $20 billion in the US [13] and over €30 billion in Europe [14,15]. Given that the proportion of people aged 65 and older is increasing, this will likely lead to an even more significant burden of disease [4,15].

A substantial body of evidence supports the use of various interventions for the detection and treatment of osteoporosis in high risk patients, and the prevention of related fractures including therapy with calcium, vitamin D, and drugs that decrease bone resorption or increase bone formation [16]. For example, bisphosphonates reduce the risk of future osteoporosis-related fracture by 40 to 60% with fracture reduction apparent within a year of starting treatment [16].

Despite the incorporation of this evidence from randomized trials into clinical practice guidelines, these interventions are considerably underutilized [17-23]. A systematic review found that the proportion of individuals with a fragility fracture who received a diagnostic test for osteoporosis or a diagnosis from a physician ranged from 1.7% to 50% [18]. Furthermore, rates of osteoporosis treatment among elderly patients with a fracture of the wrist, hip or vertebrae are less than 10 to 20% in the year following fracture [18].

These care gaps highlight the finding that additional effort is needed to ensure that appropriate knowledge translation is achieved to optimize management of osteoporosis in patients at risk of fragility fractures. This study was designed to help fill this knowledge to practice gap. The primary objective of this study was to determine if a multicomponent, community-based strategy could optimize the evidence-based management of people at risk for osteoporosis-related fractures.

## Methods

Between March 2003 and January 2006, we conducted a randomized trial of a multifaceted community based intervention to optimize care of patients at risk for osteoporosis-related fractures. The complete study protocol has been published [24].

### Setting and Study Population

This was a population-based study completed in the Algoma District of Ontario, Canada, a geographically vast area with Sault Ste Marie (population 78 000) as its main city. The study represented a partnership among consumers, providers and other stakeholders interested in reducing the evidence to practice gap. It was conducted by the Group Health Centre (GHC), a not-for-profit health service community centre, in partnership with Sault Area Hospital (SAH), a facility with 250 active beds.

Patients were eligible for inclusion in the study if they were community-dwelling, aged 55 years or older, able to give informed consent, and were identified to be at risk for future fracture according to one of the following criteria:

1. attended the hospital Fracture Clinic for a non-pathological fracture of the vertebrae, hip or wrist or had a BMD in the past year with a T-score of ≤-2.0

2. attended the hospital Emergency Department with a fall and found to be at high risk for falls as defined by a Timed Up and Go [25] result of greater than 14 seconds; or,

3. were self-referred or referred by a health care provider because of perceived high risk of fracture and identified as a high risk for falls defined by a Timed Up and Go result of greater than 14 seconds.

Patients already receiving appropriate pharmacological therapy for osteoporosis as outlined in the Osteoporosis Canada guidelines [16] were excluded from the study.

### Intervention

The intervention was multifaceted and consisted of providing evidence-based management strategies for osteoporosis to both the patients and their primary care providers. Following randomization, a bone mineral density (BMD) test was facilitated for participants during the intervention if it had not been done within the past year, and the results were sent to the primary care physician along with relevant prescribing information based on the Osteoporosis Canada guidelines [16]. A complete list of the patient's medications was compiled from two sources and provided to physicians: (1) the patient's pharmacy records; and (2) home visits conducted by the study nurses. Medications associated with increased risk of fracture were identified, and physicians were asked to assess this list of flagged medications (Appendix). The nurse assessed participants allocated to the intervention group in their home and completed the Berg Balance Scale [26], the InterRAI Screener [27,28], a medication review and an assessment for orthostatic hypotension

Patients received personalized counseling from the research nurse about osteoporosis, including a written summary of the proposed management plan. They also received educational materials on calcium and vitamin D, risk factors for osteoporosis, and their BMD results. For those few individuals without a primary care physician, the same materials were provided but patients were encouraged to visit a walk-in clinic or the emergency department.

### Allocation and Blinding

Eligible patients were randomized using a computer generated randomization scheme under supervision of the study biostatistician, into an immediate intervention protocol (IP) group or to usual care. 6 months after randomization participants in the usual care group received the intervention and were thus described as the delayed intervention protocol (DP) group. Patients, treating physicians and outcomes assessors could not be blinded to the fact that patients were participating in an osteoporosis improvement study. All patients were followed-up at 6 and 12 months after the completion of the initial assessment.

### Outcomes and Data Collection

The primary outcome under investigation was appropriate osteoporosis management based on the 2002 clinical practice guidelines for osteoporosis in Canada [16]. This was the most current evidence-based guideline available for use at the time of study onset and was generally consistent with current US and UK guidelines. Measurements of outcomes were obtained through patient records (obtained through the Electronic Medical Record) and pharmacy data. A standardized chart review of the Electronic Medical Record was the primary source of data for both groups.

Secondary outcomes included appropriate osteoporosis management at 12 months and disease-specific quality of life measured using the OPTQoL [29], a quality of life questionnaire for people with osteoporosis.

### Sample size and Analysis

Local pilot data suggested that 40% of people would receive appropriate osteoporosis medications at 6 months. We determined a minimal clinically important difference of 20% and with a two-tailed alpha of 0.05, power of 0.80, the patient as the unit of allocation and analysis, and assuming a loss to follow-up of 10%, a total sample size of 200 patients was needed.

Relative risks with 95% confidence intervals were calculated to assess outcomes between the IP group and the usual care group at 6 months. Fisher's Exact Test was used to evaluate differences in outcomes between the IP group and the DP group at 12 months. Paired t-tests were used to detect differences in the OPTQoL at baseline and 6 months within each group. Analysis was by intention to treat.

Ethics approval was received from the Joint Group Health Centre/Sault Area Hospital Research and Ethics Board. This trial has been registered with http://clinicaltrials.gov (ID: NCT00465387)

## Results

### Patient characteristics

**Figure 1:**
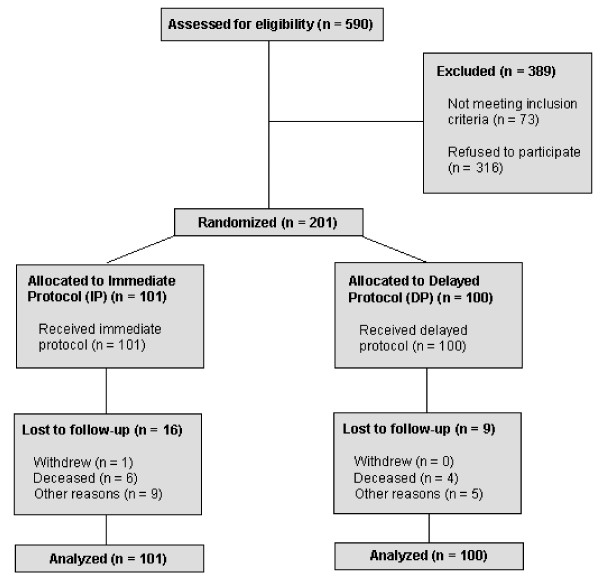
**Flow of Trial Participants**.

590 patients were screened for eligibility, 389 were excluded and 201 patients were recruited from March 2003 to January 2006, with 101 patients randomized into the IP group, and 100 patients into the usual care group (Figure 1). One hundred seventy six patients (88%) completed the study. Mean age of participants was 71.9 ± 7.2 years and 94% were women. Most participants entered the study on the basis of a previous BMD test or referral from fracture clinic suggesting that the study population was at high risk of osteoporosis-related fracture. Baseline characteristics were similar among the groups (Table 1). No statistically significant baseline differences were detected between groups with respect to presence of risk factors or differences in losses due to death and follow-up.

**Table 1:** Baseline characteristics

	*N *= 201	
Characteristic	IP (*n *= 101)	DP (*n *= 100)
**Demographic, *n *(%)**		
Mean age (years) ± SD	72 ± 9.1	71 ± 7.7
Female	92 (91.1%)	96 (96.0%)
Male	9 (8.90%)	3 (3.00%)
Aboriginal Origin	2 (1.98%)	9 (9.00%)
**Referral Source, *n *(%)**		
#1 SAH Fracture Clinic or BMD t-score ≤-2.0	74 (72.6%)	73 (73.0%)
#2 SAH Emergency Department	4 (3.96%)	5 (5.00%)
#3 Self-referred or referred by health care provider and Timed Up and Go (TUG) >14 s	23 (22.8%)	22 (22.0%)
Osteoporosis Risk Factors, *n *(%)*		
T-score +2.5 to -1.0	10 (9.90%)	9 (9.00%)
T-score -1.0 to -2.5	39 (38.6%)	31 (31.0%)
T-score ≤ -2.5	52 (51.5%)	60 (60.0%)

Note: All participants had a BMD completed as per protocol.

*No significant differences were detected between groups

### Primary Outcomes

#### Osteoporosis risk management

For osteoporosis risk management (Table 2), more than twice the number of patients with osteoporosis (BMD T-score ≤ -2.5) were taking alendronate, risedronate, or raloxifene in the IP group after 6 months compared to the usual care group (29/52 vs. 16/60; relative risk [RR] 2.09, 95% confidence interval (CI) 1.29 to 3.40). The difference in treatment rates was even greater for patients with low bone mass (T-score between -2.5 and -1.0) between the IP and usual care groups (16/39 vs. 5/31; RR 2.54, 95% CI 1.05 to 6.17). A higher proportion of patients in the IP group were using calcium (54/101 vs. 20/100; RR 2.67, 95% CI 1.74 to 4.12) and vitamin D (33/101 vs. 20/100; RR 1.63, 95% CI 1.01 to 2.65) at 6 months compared to the usual care group. There were more women in the IP group taking estrogen/progestin compared to the usual care group at 6 months (13/101 vs. 2/100; RR 6.77, 95% CI 1.49 to 27.8).

**Table 2:** Management of osteoporosis risk from randomization date until 6 months after enrolment

	*N *= 201			
Variable, *n *(%)	IP (*n *= 101)	DP (*n *= 100)	RR (95% CI of RR)	DP 6-12 months* (*n *= 100)
**Appropriate osteoporosis risk management**				
Use of alendronate, risedronate, raloxifene with BMD result of t ≤ -2.5 (osteoporosis)	29/52 (55.8%)	16/60 (26.7%)	2.09 (1.29,3.40)	33/60 (55.0%)
Use of alendronate, risedronate, raloxifene with BMD result of -2.5 < t < -1 (osteopenia)	16/39 (41.0%)	5/31 (16.1%)	2.54 (1.05,6.17)	8/31 (25.8%)
Recommended amount of calcium	54/101 (53.5%)	20/100 (20.0%)	2.67 (1.74,4.12)	46/100 (46.0%)
Recommended Amount of vitamin D	33/101 (32.7%)	20/100 (20.0%)	1.63 (1.01,2.65)	36/100 (36.0%)
Estrogen or progestin	13/101 (12.9%)	2/100 (2.00%)	6.44 (1.49,27.8)	6/100 (6.00%)
**Health Status**				
Mean OPTQoL score ± SD at baseline	205 ± 58	212 ± 54	N/A	
Mean OPTQoL score ± SD after 6 mos. of intervention	207 ± 55	220 ± 50		
P-value of difference	0.58	0.26		

Note: IP = immediate intervention protocol; DP = delayed intervention protocol;

RR = relative rate; 95% CI = 95% confidence interval

*No significant differences detected between IP and DP at 6-12 months for osteoporosis risk management variables

### Secondary Outcomes

#### Osteoporosis management

Following intervention in both groups at 12 months after randomization, the use of appropriate treatments for osteoporosis (alendronate, risedronate, raloxifene with BMD T-score ≤ -2.5) reached 56%. The usual care group improved to the same level of treatment with alendronate, risedronate, raloxifene, calcium, and vitamin D after receiving the intervention (*P = *0.48) (Table 2).

#### Quality of life and other patient-centered outcomes

Quality of life measured by the OPTQoL questionnaire remained similar within the IP group (*P *= 0.58) and DP group (*P = *0.26) over the course of the study (Table 2). One participant in the IP group had a fragility fracture at 6 months compared with 6 participants in the usual care group (RR 0.17, 95% CI 0.02,1.35). Two individuals in the IP group were admitted to hospital with a fall-related injury compared with 3 in the usual care group (RR 0.66, 95% CI 0.11 to 3.87).

## Discussion

This study demonstrated that a multi-component intervention program directed at patients at risk for fracture and at their physicians doubled the rate of appropriate osteoporosis management compared to usual care in this community. The co-ordinated intervention increased rates of osteoporosis medication use by more than 20% over usual care, a consensus benchmark previously reported as what might be considered a clinically important benefit for such an intervention [30]. Our study increased pharmacological treatment of osteoporosis by 29% over local usual care (IP 56% vs. DP 27%). Similarly, a controlled trial by Majumdar et al reported appropriate use of osteoporosis medications to be 40% in the intervention group versus 10% in the usual care control, but only patients with a wrist fracture were eligible for inclusion [31]. A more recent trial of a case manager for patients with hip fracture found similar results to what was observed in our study [32]. With the improvements in care delivery observed in the current study, the appropriate osteoporosis treatments were utilized in over one-half of individuals with receipt of the intervention. This result is better than that achieved in most other studies of interventions to enhance implementation of osteoporosis management in broad populations of at-risk patients rather than restricted to patients with recent fracture. Randomized intervention studies directed at physicians and/or patients at high risk of osteoporosis-related fractures conducted by Solomon and colleagues [33,34] and by Elston Lafata and colleagues [35] found that screening and treatment rates were either unaffected [33,34] or improved by less than 10% [35]. And a recent study of a falls-and-fracture nurse coordinator found that neither falls nor osteoporosis management were enhanced with this intervention [36]. Thus, the present study achieved an effect size substantially larger than recent studies of similar design.

The strengths of this study included a randomized study design; an intervention targeted at high-risk patients; the successful partnership between numerous distinct community stakeholders; and that we were able to 'replicate' the intervention's effects 6 months later in the DP group. Moreover, multiple health care providers and community health service groups collaborated in a coordinated assessment and treatment program to deliver evidence-based and timely interventions. Economic analysis of this study is currently underway.

The limitations of this study included: a short follow-up period of 6 months, which did not allow for longer-term results such as fracture data; and, no blinded outcomes assessors. The lack of blinding of the outcomes assessors could result in bias such as overestimation of the impact of the intervention. However, the primary source of data collection was the administrative data obtained from the Group Health Centre Electronic Medical Record. Even if we consider the lower limit of the confidence interval (for use of appropriate pharmacological therapy [RR] 2.09, 95% **CI 1.29 **to 3.40) as the effect size, we consider this a clinically important change. Given that there is evidence that appropriate management of osteoporosis decreases fractures, it was felt that the use of appropriate management as the primary outcome was relevant. Indeed, when the evidence-base is well-established (such as in osteoporosis management), process-of-care measures are more sensitive to changes in quality of care than outcomes such as mortality or clinical events and are widely recognized as acceptable and appropriate endpoints for quality improvement studies.

## Conclusions

In summary, it is critical that the health care community address the deficiencies that exist with respect to knowledge translation and management of osteoporosis given the significant burden of disease related to fractures. This randomized, community-based study supports implementation of a co-ordinated osteoporosis management strategy for improved care in at-risk individuals, although long-term follow-up should be considered. Many of the assessment and treatment protocols used in this study could be employed in existing clinics and programs to enhance osteoporosis care in a community setting.

## Competing interests

The authors declare they have no competing interests. While the study was funded as noted in the next section, none of the authors have received funds (e.g. honoraria, travel support etc) from any of these funders.

## Appendix: Risk Medications

Medications associated with an increased risk of osteoporosis:

1. *Glucocorticoids: *Cortef, cortisone, dexamethasone, hydrocortisone, prednisone, betamethasone, fludrocortisone, methylprednisone, prednisolone, tramcinolone.

2. *Inhaled Corticosteroids: *beclomethasone, budesonide, fluticasone, flunisolide, tramcinolone.

## Authors' contributions

PMC and HL were the principal investigators. After the death of HL, SES assisted with study completion. PMC, SES, LRD, RAG, KML, CRW, SRM, and SS contributed to the design, analysis and interpretation of the study as well as the drafting of this manuscript. RAG, GMZ, LAF completed the analysis for the study. SES is the guarantor for this paper. All authors read and approved the final version of the manuscript.

## Pre-publication history

The pre-publication history for this paper can be accessed here:

http://www.biomedcentral.com/1471-2318/10/60/prepub

## References

[B1] Gullberg B Johnell O Kanis JA World-wide projections for hip fracture Osteoporosis Int 1997 7 407 13 10.1007/PL00004148 9425497

[B2] Lane NE Epidemiology, etiology, and diagnosis of osteoporosis American Journal of Obstetrics and Gynecology 2006 194 S3 11 10.1016/j.ajog.2005.08.047 16448873

[B3] National Osteoporosis Society http://www.nos.org.uk/Accessed on June 22, 2010

[B4] Osteoporosis Canada http://www.osteoporosis.ca/english/home/Accessed on Je 22, 2010

[B5] Papaioannou A Adachi JD Parkinson W Stephenson G Bedard M Lengthy hospitalization associated with vertebral fractures despite control for comorbid conditions Osteoporos Int 2001 12 10 870 874 10.1007/s001980170039 11716191

[B6] Wiktorowicz ME Goeree R Papaioannou A Adachi JD Papadimitropoulos E Economic implications of hip fracture: health service use, institutional care and cost in Canada Osteoporos Int 2001 12 4 271 278 10.1007/s001980170116 11420776

[B7] Ioannidis G Papaioannou A Hopman WM Akhtar-Danesh N Anastassiades T Pickard L Kennedy CC Prior JC Olszynski WP Davison KS Goltzman D Thabane L Gafni A Papadimitropoulos EA Brown JP Josse RG Hanley DA Adachi JD Relation between fractures and mortality: results from the Canadian Multicentre Osteoporosis study CMAJ 2009 181 5 265 271 19654194 10.1503/cmaj.081720 PMC2734204

[B8] Melton LJ Chrischilles EA Cooper C Lane AW Riggs BL Perspective: how many women have osteoporosis? J Bone Miner Res 1992 7 1005 10 10.1002/jbmr.5650070902 1414493

[B9] Cummings SR Black DM Rubin SM Lifetime risks of hip, colles' or vertebral fracture and coronary heart disease among white postmenopausal women Arch Int Med 1989 149 2445 8 10.1001/archinte.149.11.2445 2818106

[B10] Melton LJ Who has osteoporosis? J Bone Miner Res 2000 15 2309 14 10.1359/jbmr.2000.15.12.2309 11127196

[B11] Chrischilles EA Butler CD Davis CD Wallace RB A model of lifetime osteoporosis impact Arch Intern Med 1991 152 2026 32 10.1001/archinte.151.10.2026 1929691

[B12] Goeree RG O'Brien B Pettitt DB Cuddy L Ferraz M Adachi JD An assessment of the burden of illness due to osteoporosis in Canada J Soc Obstet Gynaecol Can 1996 18 suppl 15 24

[B13] Cummings SR Melton LJ Epidemiology and outcomes of osteoporotic fractures The Lancet 2002 359 1761 7 10.1016/S0140-6736(02)08657-9 12049882

[B14] Poole KES Compston JE Clinical review: Osteoporosis and its management BMJ 2006 333 1251 6 10.1136/bmj.39050.597350.47 17170416 PMC1702459

[B15] International Osteoporosis Foundation http://www.iofbonehealth.org/facts-and-statistics.html Accessed on August 24, 2010

[B16] Brown JP Josse RG for the Scientific Advisory council of the Osteoporosis Society of Canada. 2002 clinical practice guidelines for the diagnosis and management of osteoporosis in Canada CMAJ 2002 167 10 suppl S1 34 12427685 PMC134653

[B17] Port L Center J Briffa NK Nguyen T Cumming R Eisman J Osteoporotic fracture: missed opportunity for intervention Osteoporos Int 2003 14 780 4 10.1007/s00198-003-1452-x 12904835

[B18] Papaioannou A Giangregorio L Kvern B Boulos P Ioannidis G Adachi JD The osteoporosis care gap in Canada BMC Musculoskel Disorders 2004 5 11 10.1186/1471-2474-5-11 PMC420244 15068488

[B19] Feldstein AC Nichols G Elmer P Smith DH Aickin M Herson M Older women with fractures: Patients falling through the cracks of guideline-recommended osteoporosis screening and treatment J Bone Joint Surg 2003 85 2294 2302 14668497

[B20] Castel H Bonneh DY Sherf M Liel Y Awareness of osteoporosis and compliance with management guidelines in patients with newly diagnosed low-impact fractures Osteoporos Int 2001 12 559 64 10.1007/s001980170077 11527053

[B21] Feldstein A Elmer PJ Orwall E Herson M Hillier T Bone mineral density measurement and treatment for osteoporosis in older individuals with fractures Arch Int Med 2003 163 2165 72 10.1001/archinte.163.18.2165 14557214

[B22] Giangregorio L Papaioannou A Cranney A Zytaruk N Adachi JD Fragility fractures and the osteoporosis care gap: an international phenomenon Semin Arthritis Rheum 2006 35 5 293 305 10.1016/j.semarthrit.2005.11.001 16616152

[B23] Elliot-Gibson V Bogoch ER Jamal SA Beaton DE Practice patterns in the diagnosis and treatment of osteoporosis after a fragility fracture: a systematic review Osteoporos Int 2004 15 767 778 10.1007/s00198-004-1675-5 15258724

[B24] Ciaschini P Straus SE Dolovich LR Goeree RA Leung KM Woods CR Zimmerman GM Majumdar SR Spadafora S Fera LA Lee HN Management of patients at risk for osteoporosis: a randomised trial Trials 2008 9 1 62 10.1186/1745-6215-9-62 18983670 PMC2612651

[B25] Shumway-Cook A Brauer S Woollacott M Predicting the probability for falls in community-dwelling older adults using the Timed Up and Go Test Phys Ther 2000 80 896 903 10960937

[B26] Berg KO Wood-Dauphinee SL Williams JT Maki B Measuring balance in the elderly: validation of an instrument Can J Public Health 1992 83 S7 11 1468055

[B27] InterRAI.org [homepage on the Internet] http://www.interrai.org/section/view/?fnode=14 Ann Arbor: University of Michigan Institute of Gerontology; [cited 2006 Oct 21]. InterRAI screener; [about 1 screen]

[B28] Shumway-Cook A Baldwin M Polissar N Gruber W Predicting the probability for falls in community-dwelling older adults Phys Ther 1997 77 812 9 9256869 10.1093/ptj/77.8.812

[B29] Lydick E Zimmerman SI Yawn B Love B Kleerekoper M Ross P Martin A Holmes R Development and validation of a discriminative quality of life questionnaire for osteoporosis J Bone Miner Res 1997 12 456 63 10.1359/jbmr.1997.12.3.456 9076589

[B30] Hajcsar EE Hawker G Bogoch ER Investigation and treatment of osteoporosis in patients with fragility fractures CMAJ 2000 163 7 819 822 11033708 PMC80503

[B31] Majumdar SR Rowe BH Folk D Johnson JA Holroyd BH Morrish DW Maksymowych WP Steiner IP Harley CH Wirzba BJ Hanley DA Blitz S Russell AS A controlled trial to increase detection and treatment of osteoporosis in older patients with a wrist fracture Ann Intern Med 2004 141 366 373 15353428 10.7326/0003-4819-141-5-200409070-00011

[B32] Majumdar SR Beaupre LA Harley CH Hanley DA Lier DA Juby AG Maksymowych WP Cinats JG Bell NR Morrish DW Use of a case manager to improve osteoporosis treatment after hip fracture Arch Intern Med 2007 167 19 2110 5 10.1001/archinte.167.19.2110 17954806

[B33] Solomon DH Polinski JM Stedman M Truppo C Breiner L Egan C Jan S Patel M Weiss TW Chen YT Brookhart MA Improving care of patients at risk for osteoporosis: a randomized controlled trial JGIM 2007 22 362 367 10.1007/s11606-006-0099-7 17356969 PMC1824772

[B34] Solomon DH Katz JN Finkelstein JS Polinski JM Stedman M Brookhart MA Arnold M Gauthier S Avorn J Osteoporosis improvement: a large scale randomised controlled trial of patient and primary care physician education J Bone Miner Res 2007 22 1808 15 10.1359/jbmr.070717 17645403

[B35] Elston Lafata J Kolk D Peterson EL McCarthy BD Weiss TW Chen YT Muma BK Improving osteoporosis screening: results from a randomized cluster trial JGIM 2007 22 346 51 10.1007/s11606-006-0060-9 17356966 PMC1824751

[B36] Elley CR Robertson MC Garrett S Kerse NM McKinlay E Lawton B Moriarty H Moyes SA Campbell AJ Effectiveness of a falls-and-fracture nurse coordinator to reduce falls J Am Geriatr Soc 2008 56 1383 9 10.1111/j.1532-5415.2008.01802.x 18808597

